# Bayley's Pocket-Book for Pharmacists
*"Bayley's Pocket Book for Pharmacists." (London: Messrs. E. and F. N. Spon.)


**Published:** 1890-08-30

**Authors:** 


### THE PRACTITIONER'S BOOKSHELF.

[Books for Review should be sent to The Editor, The Lodge, Porchester Square, W.]

### BAYLEY'S POCKET-BOOK FOR PHARMACISTS.*

This convenient little compilation is likely to meet a want. It first gives the natural history, names, doses, uses, and tests of the drugs of the British and American Pharmacopœias. Then those of the substances not found in the B.P., but existing in the United States Pharmacopœia. After follow useful notes on ordinary food materials, condiments, beverages, &c. Then an alphabetical list of pharmaceutical preparations, with a long series of tables of the same from both pharmacopœias. Next a useful list of American Eclectic Resinoids. A note at the end of these should be remembered: "These so-called active principles are precipitated by pouring a concentrated alcoholic fluid extract into water. These must not be confounded with the active principle, although the names are often exactly the same." That is, don't mix up the precipitated spirituous extract of the drug with the alkaloid of the same. A table showing the botanical derivation of the official drugs follows, with a fairly complete botanical glossary; one of the chief Latin terms used in pharmacy; and a long one of terms used in medicine, not including the names or classes of drugs. A good resumé of the domestic and general treatment of poisons is followed by tables of solubility of substances in water and other liquids, and by numerous other tables for the benefit of apothecaries and pharmaceutical chemists. The book concludes with a list of general recipes. There are a few blank pages at either end of the book for the purposes of note taking.

We think Mr. Bayley has succeeded in producing a distinctly original and valuable combination of materials in the somewhat well-worn and oftentimes tedious paths of *materia medica*. The book is small and convenient to the hand, the type clear, and the arrangement of subjects easy to work with. We wish the book every success.

* "Bayley's Pocket Book for Pharmacists." (London: Messrs. E. and F. N. Spon.)

